# Comparisons of the iron deficient metabolic response in rats fed either an AIN-76 or AIN-93 based diet

**DOI:** 10.1186/1743-7075-9-95

**Published:** 2012-10-30

**Authors:** McKale R Davis, Kristen K Hester, Krista M Shawron, Edralin A Lucas, Brenda J Smith, Stephen L Clarke

**Affiliations:** 1 Department of Nutritional Sciences, Oklahoma State University, Stillwater, OK 74078, USA

**Keywords:** Hyperglycemia, Lipogenesis, Insulin, Metabolism, Iron deficiency

## Abstract

**Background:**

Previous studies examining the metabolic consequences of dietary iron deficiency have reported elevated serum glucose concentrations in iron-deficient animals. Importantly, the majority of these findings were observed using an earlier version of a laboratory animal diet (AIN-76A) in which the primary carbohydrate source was sucrose – a disaccharide known to negatively impact both glucose and lipid homeostasis. The AIN-76A diet formula was improved in 1993 (AIN-93) to optimize animal nutrition with a major change being the substitution of cornstarch for sucrose. Therefore, we sought to examine the effects of iron deficiency on steady-state glucose homeostasis and the hepatic expression of glucose- and lipid-related genes in rats fed an iron-deficient diet based on either an AIN-76A or AIN-93 diet.

**Methods:**

The study design consisted of 6 treatment groups: control (C; 40 mg Fe/kg diet), iron deficient (ID; ≤ 3 mg Fe/kg diet), or pair-fed (PF; 40 mg Fe/kg) fed either an AIN-76A or AIN-93 diet for 21 d. Hemoglobin and hematocrit were measured in whole blood. Serum insulin and cortisol were measure by ELISA. Serum glucose and triacylglycerols were measured by standard colorimetric enzyme assays. Alterations in hepatic gene expression were determined by real-time qPCR.

**Results:**

Hemoglobin and hematocrit were significantly reduced in both ID groups compared to the C and PF groups. Similarly, animals in the both ID groups exhibited elevated steady-state levels of blood glucose and insulin, and significantly decreased levels of circulating cortisol compared to their respective PF controls. Serum triacyglycerols were only increased in ID animals consuming the AIN-76A diet. Hepatic gene expression analyses revealed a ~4- and 3-fold increase in the expression of glucokinase and pyruvate dehydrogenase kinase-4 mRNA, respectively, in the ID group on either diet compared to their respective PF counterparts. In contrast, the expression of lipogenic genes was significantly elevated in the AIN-76 ID group, while expression of these genes was unaffected by iron status in the AIN-93 ID group.

**Conclusions:**

These results indicate that an impaired iron status is sufficient to alter glucose homeostasis, though alterations in lipid metabolism associated with ID are only observed in animals receiving the AIN-76A diet.

## Background

Iron is an essential nutrient found in abundance in the earth’s crust, yet iron deficiency remains the most common micronutrient deficiency in the world [1]. Symptoms of iron deficiency include weakness, fatigue, impaired immune function, and reduced cognitive function in children. In animal models, less well characterized responses to iron deficiency include alterations in lipid and glucose metabolism arising from decreased oxidative capacity which leads to a shift in preferential fuel utilization from fat to glucose [2-4]. Iron-deficient animals also display signs of disrupted metabolic homeostasis, including alterations in insulin signaling, as evidenced by hyperglycemia, hyperinsulinemia, and hyperlipidemia [2,5].

Determining the molecular mechanisms contributing to the hyperglycemic and hyperinsulinemic responses observed in response to dietary iron deficiency is made more complicated since many of the observations in glucose metabolism were noted prior to the 1993 American Institute of Nutrition (AIN) reformulation (AIN-93) of the AIN-76A laboratory animal diet, which is simply referred to as AIN-76 in the present study. The AIN-93 rodent diets were formulated to improve animal performance in experimental models with a major change being the substitution of cornstarch for sucrose because high dietary concentrations of sucrose were associated with several metabolic complications including hyperlipidemia, hyperinsulinemia, and fatty liver [6,7]. Therefore, it is difficult to discern the extent to which the metabolic consequences observed in previous studies noting disruptions in metabolic homeostasis were indeed an effect of iron deficiency alone, or rather the effect of dietary carbohydrate (i.e., sucrose) on glucose and lipid metabolism in iron-deficient animals.

Another factor complicating the investigation into the metabolic response to iron deficiency is that the severity of these consequences (i.e., hyperglycemia and hyperlipidemia) appears to be a graded response associated with a reduction in hemoglobin [8]. Hemoglobin levels indicative of anemia are associated with elevated plasma triacylglycerols (TAG) and glucose, though less severe reductions in hemoglobin are not as highly correlated with hyperlipidemia and hyperglycemia suggesting that a certain threshold exists in order to develop these potentially negative metabolic consequences [8-10]. In contrast, neither hyperglycemia nor hyperlipidemia were observed at varying levels of anemia in animals fed an AIN-93 based diet, although both glucose utilization and insulin responsiveness appeared to be enhanced [2]. More recently, two studies reported elevated serum glucose and TAG levels in severely iron-deficient (hemoglobin < 60 g/L) rodents fed an AIN-93 diet, but the underlying mechanisms contributing to these metabolic responses were not the primary focus of these investigations [11,12]. Thus, it remains unclear if these mixed observations from previous studies are more attributable to the severity of iron deficiency elicited in the animal model, or are instead the result of a carbohydrate-specific response to an iron-deficient diet.

The focus of the current study was to examine the extent to which an impaired iron status is associated with alterations in metabolic homeostasis and changes in hepatic lipogenic gene expression in rats fed iron-deficient diets based on either the AIN-76 or AIN-93 formulations. Animals in the iron-deficient groups, regardless of carbohydrate source, exhibited elevated levels of steady-state serum glucose and insulin. Interestingly, serum TAG and the relative abundance of mRNA encoding proteins responsible for regulating *de novo* lipogenesis in the liver was increased only in iron-deficient rats receiving the AIN-76 diet. The results presented herein support a model wherein alterations in glucose homeostasis observed in iron-deficient animals are independent of dietary carbohydrate whereas alterations in lipid metabolism appear to be dependent of dietary carbohydrate present in the AIN-76 diet. Thus, it is essential to consider the metabolic consequences of diets used to study the effects of micronutrient deficiencies in animal models.

## Methods

### Study design

Forty-eighty 21-d-old weanling male Sprague–Dawley (Harlan, IN) rats were housed individually at the Oklahoma State University (OSU) Laboratory Animal Research facility in a temperature- and humidity-controlled environment and maintained on a 12 h light:dark cycle with *ad libitum* access to deionized water. Upon arrival at the animal facility, rats were randomly assigned to either the AIN-76 (n=24) or AIN-93G (n=24) arm of the study (Table 1). Rats in each group were allowed *ad libitum* access to their respective control diet for 3 d prior to starting dietary treatments. After the acclimation period, rats in each group were assigned to one of three treatments (n=8/treatment) for 21 d: control (76-C or 93-C; 40 mg Fe/kg diet), iron-deficient (76-ID or 93-ID; ≤ 3 mg Fe/kg diet), or pair-fed (76-PF or 93-PF; each were fed their respective control (40 mg Fe/kg diet) diets at the level of intake of their ID counterparts). Commercially available powdered diets (76-C-TD.89300, 76-ID-TD.80396, 93-C-TD.94045, and 93-ID-TD.09564) were purchased from Harlan Teklad (Madison, WI). The primary differences between the AIN-76 and AIN-93 diets pertinent to the current study are shown in Table 1. Whereas cellulose was notably absent from the 93-ID in an effort to prevent iron contamination, the 93-C diet contained 50 g cellulose/kg diet. Previous studies have shown that this level of cellulose (5% w/w) does not adversely affect apparent absorption of iron [13]. Individual body weights and food intake were measured daily. After the 21 d experimental period, rats were anesthetized with a mixture of ketamine/xylazine and killed by exsanguination between 8:00 and 10:00 a.m. Food intake was not restricted prior to sacrifice. All animal handling and procedures were approved by the Institutional Animal Care and Use Committee at OSU.

**Table 1:** **Composition of AIN-76 and AIN-93 diets**^**1**^

	**AIN-76 (g/kg)**	**AIN-93 (g/kg)**
Casein	200	200
DL-Methionine	3	-
L-Cystine	-	3
Corn Starch	150	**447**
Maltodextrin	-	132
Sucrose	**550**	100
Soybean Oil	-	70
Corn Oil	50	-
Mineral Mix^2^	35	35
Vitamin Mix	10	10
Choline Bitartrate	2	2.5
TBHQ, antioxidant	-	0.014
Ethoxyquin	0.01	-

^1^The 93-C diet contained 397 g/kg corn starch with an added 50 g/kg cellulose. Cellulose was removed from the 93-ID and both AIN-76 diets to prevent contamination with additional iron.

^2^Mineral mixes were adjusted such that the ID diets contained ≤ 3 mg Fe/kg diet, and the C diets contained ~40 mg Fe/kg diet.

### Assessment of iron status

Whole blood was collected from the abdominal aorta into EDTA-coated tubes and sent to a commercial laboratory (Antech Diagnostics, Inc. Irvine, CA) for determination of hemoglobin and hematocrit. A sample of whole blood was also collected into serum tubes, allowed to clot, centrifuged at 800 x *g* for 20 min at 4°C to separate the serum, and then stored at −80°C until further analyses. Serum iron was determined using an ELAN 9000 ICP-Mass Spectrometer (PerkinElmer, Norwalk, CT). Microanalysis of non-heme iron in liver was determined as described by Rebouche *et al*. [14].

### Metabolic indices

Serum glucose was measured using a glucose oxidase kit (Sigma-Aldrich, St. Louis, MO) according to the manufacturer’s instructions, except that reaction volumes were scaled down to a final volume of 1 mL. Serum was diluted 1:80 (v/v) so that results obtained were within the detectable range of the assay and standard curve. Serum insulin (Crystal Chem, Inc., Downers Grove, IL) and cortisol (R&D Systems, Minneapolis, MN) were measured by ELISA according to the manufacturer’s instructions. Serum TAG levels were measured using a standard colorimetric assay based on the enzymatic hydrolysis of triacylglycerol to glycerol and free fatty acids by lipase (Sigma-Aldrich, St. Louis, MO). For all assays, serum samples with obvious signs of hemolysis were excluded from analyses.

### Pathway-focused PCR array and qPCR

Changes in gene expression were analyzed by a pathway-focused Glucose Metabolism PCR array (SABiosciences, Valencia, CA). Briefly, total RNA was isolated from ~ 100 mg whole liver using STAT-60 (Teltest, Inc., Friendswood, TX). RNA concentration and integrity were determined using a Nanodrop spectrophotometer (Thermo Fischer Scientific, Middletown, VA) and agarose gel electrophoresis, respectively. Total RNA was then treated with DNase I, reverse-transcribed using SuperScript II (Invitrogen, Grand Island, NY), and brought to a final volume of 120 μL. The cDNA from individual animals was used as a template for the PCR array according to the array instructions using SYBR green chemistry on an ABI 7900HT system (Applied Biosystems, Grand Island, NY). Data were analyzed using SABiosciences RT^2^ Profiler PCR Data Analysis software at http://pcrdataanalysis.sabiosciences.com/pcr/arrayanalysis.php and were considered significant at > 1.3 fold change and *P* < 0.05. Relative quantitation for each gene was determined by normalizing to 4 housekeeping genes (RPLP1, HPRT1, RPL13A, and ACTB) comparing the ID and PF groups using the 2^-∆∆Ct^ method. For gene expression analysis by qPCR using SYBR green chemistry, cDNA was prepared as described above. Primers for qPCR were designed using Primer Express v 2.0 (Applied Biosystems) and validated if they met the following criteria: (1) single peak on dissociation curve and (2) amplification efficiency slope of −3.3 using titrated standard curve. Additionally, whenever possible, primers were designed such that the amplicon spanned at least one intron. Relative quantitation for each gene was determined using the 2^-∆∆CT^ method (Applied Biosystems User Bulletin #2) with Cyclophilin B (Cyclo) as the invariant control. Primer sequences used in these studies are listed in Additional file 1: Table S1.

### Statistical analysis

Comparisons of body weight, hemoglobin, hematocrit, and array data within and between treatment groups were analyzed using one-way ANOVA followed by Least Significant Difference as the *post hoc* test using SPSS software version 17.0 (IBM-SPSS, Chicago, IL). Differences in gene expression between ID and PF groups within each diet were determined using a Student’s t-test. All tests were conducted at the 95% confidence interval and presented as means ± SEM.

## Results

### Body weight and food intake

**Figure 1:**
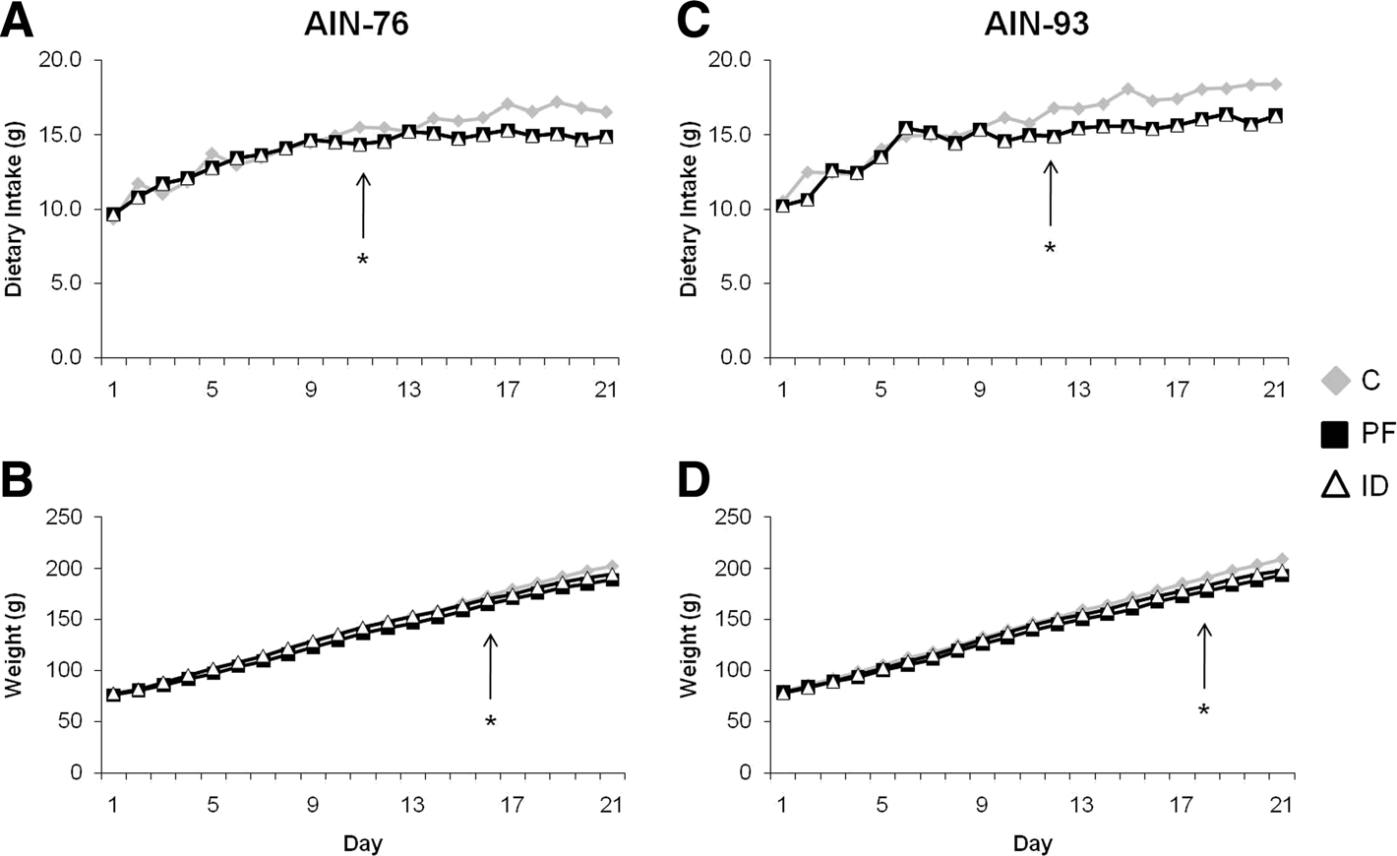
**Body weights and food intake were monitored throughout the 21 d experimental period.** Both the ID and PF groups gained significantly less weight regardless of diet. *Indicates the day at which differences in food intake and weight gain became statistically significant, *p* < 0.05.

Consistent with previous observations, rats in the ID groups consumed significantly less diet and weighed significantly less than rats in the C groups regardless of diet formulation (Figure 1) [15,16]. At the end of the dietary treatment period, rats in the 76-ID group consumed 13% less diet and weighed 10% less than the 76-C group, whereas rats in the 93-ID group consumed 7% less diet and weighed 5% less than the 93-C group. There were no differences in final body weight between rats in the PF and ID groups on either diet (Figure 1). Interestingly, rats receiving either the 76-C or 76-ID diets consumed significantly less diet than those receiving the 93-C or 93-ID diet, respectively.

### Iron status

**Figure 2:**
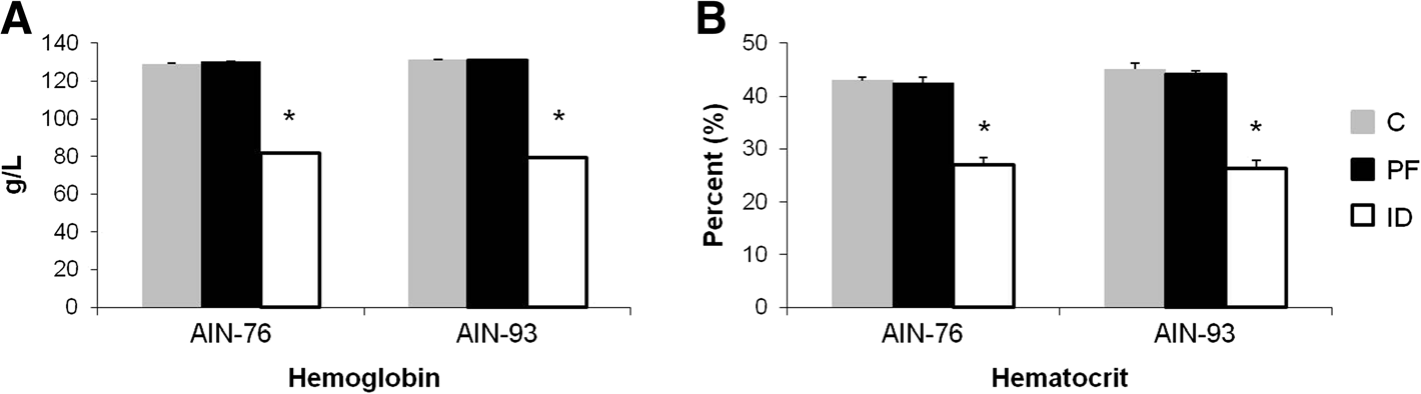
**Hematologic values indicate that only the ID animals in each group were anemic.** Levels of hemoglobin and hematocrit did not differ between AIN-76 and AIN-93 diets. Values are means ± SEM (n = 8). *Indicates statistical significances between groups, *p* < 0.05.

**Figure 3:**
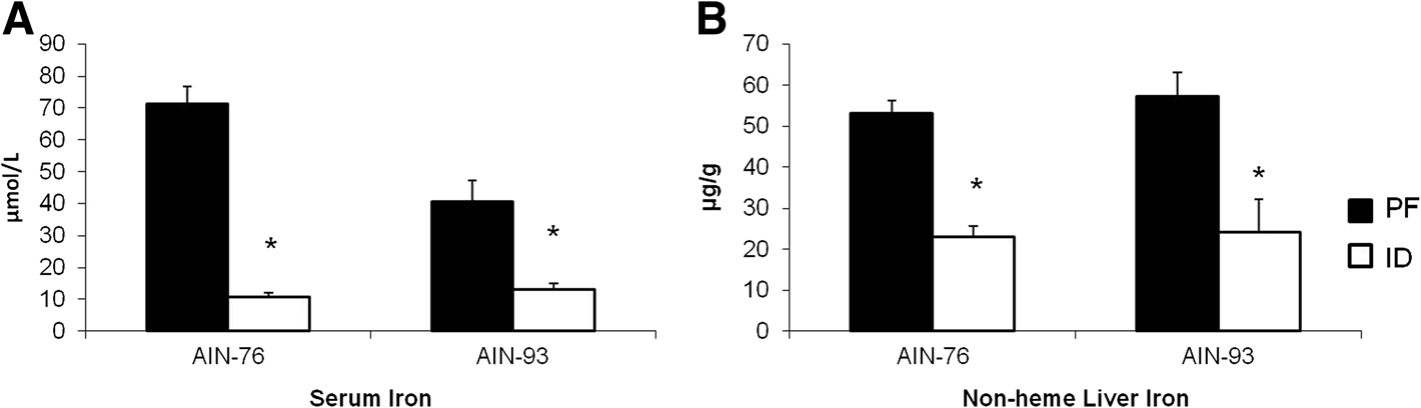
**Degrees of iron deficiency did not differ between AIN-76 and AIN-93 diets.** Serum iron and non-heme liver iron were reduced to similar degrees in ID animals fed either an AIN-76 or AIN-93 diet. Values are means ± SEM (n = 8). *Indicates statistical significance between groups, *p* < 0.05.

Importantly, only the rats in the ID groups exhibited signs of anemia as determined by hemoglobin and hematocrit (Figure 2). Regardless of diet, hemoglobin levels were decreased by ~38% in the ID groups compared to C and PF groups (Figure 2A). Similarly, rats in the 76-ID and 93-ID groups exhibited a ~37% decline in hematocrit values compared to the C and PF groups (Figure 2B). Because of the similarities in hemoglobin and hematocrit between the C and PF rats within each diet group, the remaining comparisons were made between the ID and PF groups for each diet in order to attribute any observed biological changes to a diminished iron status rather than a decrease in total nutrient intake. Lastly, serum iron and non-heme liver iron was significantly lower in the ID groups compared to the PF groups for both diets (Figure 3A-B).

It is of note that serum iron levels in the 93-PF group were significantly lower than the 76-PF group (Figure 3A). Each diet was sent to an independent laboratory (N·P Analytical Laboratories, St. Louis, MO) for analysis of iron content. The iron content of the 76-C and 93-C diets was 44 mg/kg and 41 mg/kg diet, respectively. Iron content of the 76-ID and 93-ID diets was 3 mg/kg and 2 mg/kg diet, respectively. Thus, when average daily intake of iron is compared by diet (i.e., AIN-76 *vs*. AIN-93), rats receiving the AIN-93 diet consumed less iron those consuming the AIN-76 diet, regardless of iron level in the diet (607.2 ± 25 μg/day for 76-PF *vs*. 590.8 ± 25 μg/day for 93-PF and 47.6 ± 2 μg/day for 76-ID *vs*. 31.2 ± 1 μg/day for 93-ID, *data not shown*.) Despite these differences, animals in the PF groups met the recommended guidelines for iron intake of at least 525 μg Fe/day (based on an intake of 15 g diet/day on a diet containing 35 mg Fe/kg diet) [17]. Interestingly, non-heme liver iron was not different between the 76-PF and 93-PF groups (Figure 3B), and importantly serum iron and non-heme liver iron did not differ between the 76-ID and 93-ID groups (Figure 3A-B).

### Steady-State serum levels of glucose, insulin, and triacylglycerols

**Figure 4:**
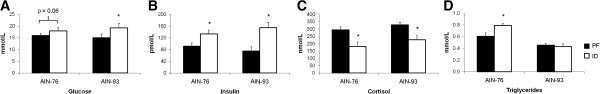
**Serum levels of glucose and insulin were significantly increased, while cortisol levels were significantly decreased in the ID groups.** ID resulted in significantly increased serum triglycerides in rats consuming the AIN-76 diet, but did not have an effect on rats consuming the AIN-93 diet. Values are given as means ± SEM (n=4-8). *Indicates statistical difference between ID and PF animals within each diet group, *p* < 0.05.

Serum glucose levels were higher than normal in all groups, but were expected as a result of the method of anesthesia used in this study [18,19]. Although not statistically significant (*P*=0.06), serum glucose in the 76-ID group tended to be higher when compared to the 76-PF group (Figure 4A). In animals receiving the AIN-93 diet, serum glucose of rats in the ID group was significantly higher than those in the 93-PF group (*P*<0.05) (Figure 4A). Serum insulin levels were increased 50% and 100% in the 76-ID and 93-ID groups, respectively, compared to their corresponding PF groups (*P*<0.05) (Figure 4B). In order to determine if elevated glucose levels were the result of increased circulating cortisol, serum cortisol was determined. Compared to their respective PF groups, serum cortisol levels were significantly decreased in the 76-ID and 93-ID groups (*P*<0.05) (Figure 4C). Intriguingly, dietary iron deficiency was only associated with elevated serum levels of TAG in rats consuming the AIN-76 diet (*P*<0.05) (Figure 4D).

### Alterations in hepatic gene expression

**Figure 5:**
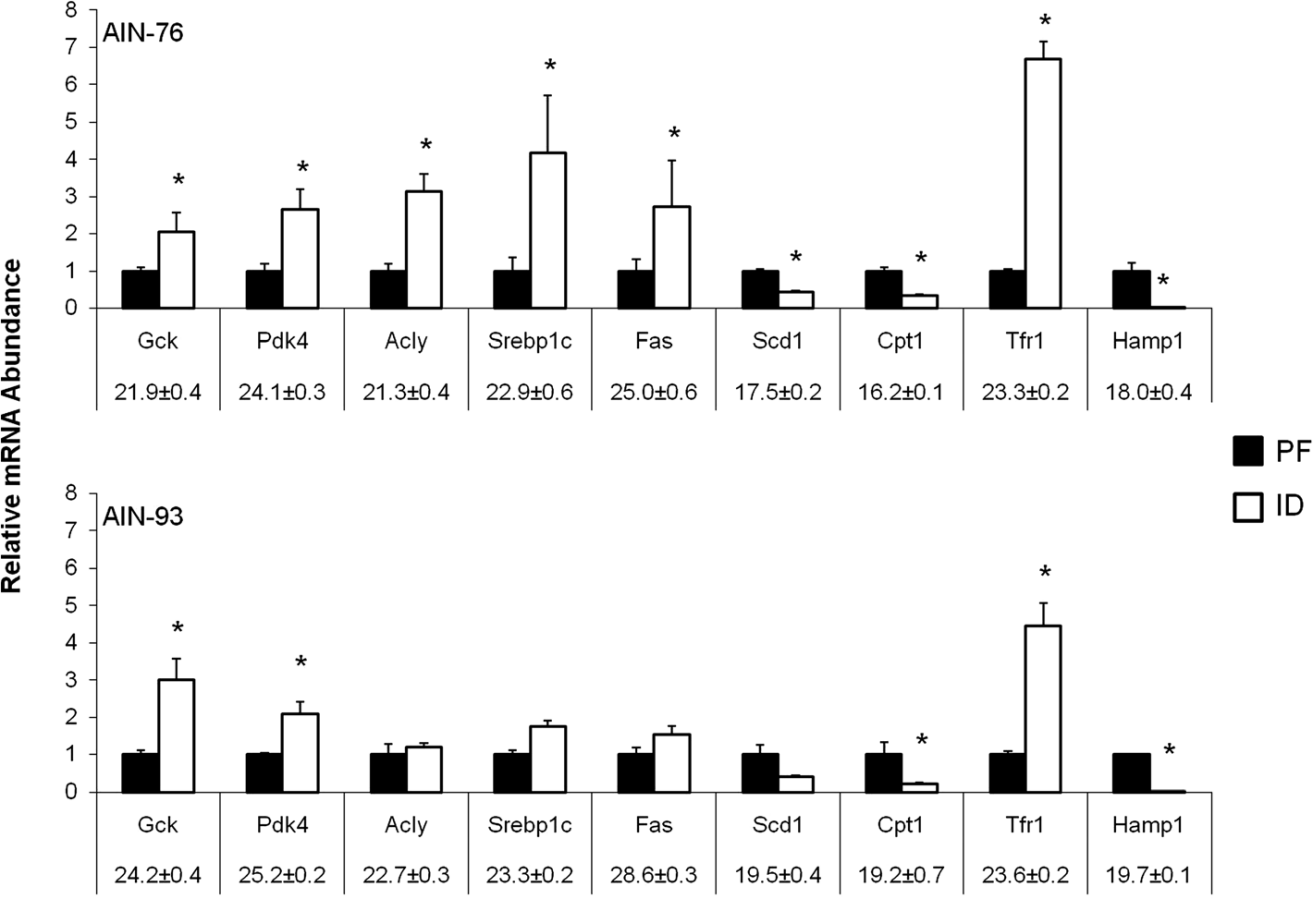
**Iron deficiency significantly affects the hepatic expression of mRNA involved in fat metabolism (*Fas*, *Acly*, *Scd1*) and insulin signaling (*Pdk4 *and *Srebp1c*).** The ID group also exhibited in significantly increased expression of *Tfr1* and significantly decreased expression of the iron sensor hepcidin (*Hamp1*). mRNA levels were normalized to Cyclophilin B (*Cyclo*) mRNA as the invariant control. Numbers beneath gene names indicate C_T_ value ± SEM obtained for the PF group. *Cyclo* Mean C_T_ = 18.9 for both PF groups. Values are given as means ± SEM (n=8). *Indicates statistical difference between ID and PF animals within each diet group, *p* < 0.05.

Using a pathway-focused PCR array approach in addition to traditional qPCR, the expression of genes involved in glucose and lipid metabolism was assessed in the livers of ID and PF animals. Relative to the PF groups, more numerous and significant changes in gene expression were detected in the 76-ID group than the 93-ID group in the PCR array (Table 2). In terms of glucose homeostasis, the most notable change in gene expression that was determined by the glucose metabolism PCR array was a ~3.5-fold increase in glucokinase (*Gck*) mRNA expression in both ID groups (Table 2). In contrast, ATP-citrate lyase (*Acly*) increased 2.9-fold in the 76-ID group compared to the 76-PF group, though similar changes were not evident in rats receiving AIN-93-based diets (Table 2). The alterations in gene expression of both *Gck* and *Acly* determined by the PCR array were further validated and confirmed by qPCR (Figure 5A-B).

**Table 2:** **Relative fold-change in mRNA abundance of genes involved in glucose metabolism**^**1**^

**Gene Name**	**Gene Symbol**	**AIN-76**	**AIN-93**
Glucokinase	Gck	+3.4	+3.9
ATP-citrate lyase	Acly	+2.9	NC
Aconitase 1 (cytosolic; IRP1)	Aco1	+1.3	+1.6
Pyruvate dehydrogenase (lipoamide) beta	Pdhb	+1.5	NC
Phosphoglucomutase 3	Pgm3	+1.5	NC
Ribose 5-phosphate isomerase A	Rpia	+1.5	+1.4
Glycogen synthase 2 (liver specific)	Gys2	−1.3	−1.6
2,3-bisphosphoglycerate mutase	Bpgm	−1.3	−1.4

^1^Values in each column indicate statistically significant relative fold-changes in mRNA abundance in each ID group compared to their respective PF group. NC is no change. Statistical significance was defined as fold-change ≥ 1.3 and *p* < 0.05.

Taking a more biased approach to assess iron- and/or diet-dependent changes in gene expression, the hepatic expression of genes involved in glucose, lipid, and iron metabolism was also examined by qPCR. The mRNA abundance of pyruvate dehydrogenase kinase-4 (*Pdk4*) was increased ~3- and 2-fold in the 76-ID and 93-ID groups, respectively (Figure 5A-B). Similar to the changes in *Acly* gene expression observed in the 76-ID group, the expression of lipogenic genes such as sterol regulatory binding protein-1c (*Srebp1c*) and fatty acid synthase (*Fas*) were also significantly increased in the livers of the 76-ID animals compared to the 76-PF animals (Figure 5A). Compared to the 93-PF group, expression of *Srebp1c* and *Fas* was not significantly different in the 93-ID group (Figure 5B). Interestingly, the expression of stearoyl CoA desaturase 1 (*Scd1*) mRNA, the rate-limiting enzyme involved in the synthesis of monounsaturated fatty acids for subsequent incorporation into TAG, was significantly decreased (~60% reduction) in the 76-ID group compared to the 76-PF group (Figure 5A). Similar results were obtained for the 93-ID group, though when compared to the 93-PF group, the results did not reach the level of statistical significance (Figure 5B). Consistent with previous findings of diminished β-oxidation activity in ID, there was significant reduction (70 – 80%) in the gene expression of carnitine palmitoyltransferase (*Cpt1*) in both ID groups regardless of diet (Figure 5A-B) [9,10,20]. Lastly, to further confirm that the livers were “sensing” iron deficiency in animals receiving an iron-deficient diet, the mRNA abundance of the iron uptake protein transferrin receptor 1 (*Tfr1*) and the iron-sensing peptide hormone hepcidin (*Hamp1*) was assessed. As expected, both the 76-ID and 93-ID groups exhibited an increased abundance (4 – 7-fold) of *Tfr1* mRNA compared to their respective PF groups (Figure 5A-B). Expression of *Hamp1* mRNA was significantly repressed (> 99% reduction) in both ID groups compared to their PF counterparts (Figure 5A-B).

## Discussion

In the current study, despite a severe dietary iron restriction, only a moderate degree of anemia (hemoglobin ~ 80 g/L) was elicited. Interestingly, even a moderate induction of iron deficiency appears to contribute to elevations in both steady-state levels of serum glucose and insulin regardless of basal diet formulation. Others have postulated that an increase in serum glucose may be due, at least in part, to an elevation of cortisol observed in severely anemic rats [21,22]. Interestingly, the relative decrease in cortisol in the ID groups in the current study suggests that other mechanisms may be responsible for the presence of hyperglycemia. In fact, the results of this study are in agreement with those obtained in a human study wherein patients with severe iron deficiency exhibited reduced cortisol secretion [23]. In addition to the relative hyperglycemia, a relative hyperinsulinemia was observed in the ID animals in both groups as well. These metabolic adaptations presumably occur as a compensatory means as ID animals preferentially utilize glucose, rather than fat, as a metabolic substrate for peripheral tissues as a result of decreased hemoglobin levels and subsequent decreased oxidative capacity [4,9,10]. Thus, blood glucose levels likely remain elevated to ensure that adequate fuel substrate is available for energy production, and insulin levels may remain elevated to facilitate the entry of glucose into insulin-dependent tissues.

In an effort then to further interrogate the underlying metabolic changes that occur with iron deficiency, we examined the hepatic expression of 84 genes involved in maintenance of glucose homeostasis. The level of significance (fold change ≥ 1.3), while small, was considered significant as previous studies have shown that dietary intervention(s) tend to elicit somewhat modest, yet biologically meaningful, transcriptional responses [24,25]. The ID rats in each group exhibited modest, but significant alterations in the expression of genes representative of glucose metabolism. Notable changes in gene expression include those genes associated with metabolic pathways including both glycolysis and gluconeogenesis and are in agreement with the findings of others [2,10,26]. The significant increase in *Gck* expression is likely due to the relative increase in circulating insulin levels observed in the ID groups, as insulin is a known inducer of hepatic *Gck* mRNA expression [27,28]. Increased expression of *Gck* could potentially be very important as ID animals have been shown to have an increased reliance on glucose as a metabolic substrate, and *Gck* is able to rapidly increase the rate of glucose phosphorylation in the liver in response to the elevations in blood glucose levels [27]. Furthermore, as Gck catalyzes the first step in hepatic glucose utilization it can contribute multiple pathways including glycogen synthesis, glycolysis, and *de novo* lipogenesis which could explain the enhanced glucose utilization and hyperlipidemia reported in response to dietary ID [2,10,26,29,30].

The significant increase in *Gck* mRNA expression, presumably in response to the elevated insulin levels, suggests that insulin signaling in ID animals is at least partially intact. However, previous observations suggest that alterations in metabolic gene expression are indicative of an impaired hepatic insulin response wherein ID animals exhibited a form of mixed insulin resistance [5]. Under normal conditions (e.g., iron sufficiency) when insulin levels are elevated in a fed state, insulin acts both to repress gluconeogenic gene expression and to simultaneously activate lipogenic gene expression [31,32]. In contrast, chronic hyperinsulinemia contributes to a combination of hepatic insulin resistance in which the insulin-dependent activation of lipogenic gene expression remains intact, but gluconeogenic gene expression is inadequately repressed [5,30,33]. In this model of mixed insulin resistance, insulin acts through the mammalian target of rapamycin complex 1 to activate lipogenesis via a *Srebp1c*-dependent increase in lipogenic gene expression, whereas insulin-induced phosphorylation of the transcription factor forkhead box protein O1 is diminished such that gluconeogenic gene expression remains inappropriately active [33]. Thus, mixed insulin resistance remains a candidate mechanism explaining the relative hyperglycemia and hyperlipidemia reported in ID animals. Despite changes in hepatic insulin signaling, peripheral tissue insulin sensitivity as assessed by glucose clearance appears to be enhanced with ID [2,4]. The extent to which there are tissue-specific differences in insulin signaling in response to iron deficiency warrants additional investigation.

To further investigate potential factors contributing to hypertriacylglycerolemia that has been reported in iron-deficient animals, changes in hepatic lipogenic gene expression were assessed in the PF and ID groups on both diets. In fact, it was the finding of increased lipogenic gene expression in iron-deficient animals consuming an AIN-76-based diet that stimulated the comparison of the AIN-76 and AIN-93 diets [5]. As early as 1982 it was speculated that causes of metabolic complications observed in animals consuming an AIN-76 diet were related to sucrose, the primary carbohydrate source in the AIN-76 formulation [34]. Not surprisingly, basal levels of the lipogenic genes *Acly* and *Fas* were higher in the 76-PF group compared to the 93-PF group, though the expression of the lipogenic master transcriptional regulator *Srebp1c* remained largely unchanged. Nonetheless, compared to the PF group, lipogenic gene expression was further significantly enhanced only in animals consuming an iron-deficient AIN-76 diet. Elevated levels of TAG in the 76-ID group suggest a functional consequence of enhanced lipogenic gene expression resulting is *de novo* fatty acid synthesis and packaging into TAG destined for secretion from the liver. Although the levels of liver TAG were not determined in the present study, taken together these data provide compelling evidence that consumption of an iron-deficient yet high-sucrose diet may result in more severe metabolic complications leading to hyperglycemia, hyperinsulinemia, and hyperlipidemia.

Despite the differences observed in lipogenic gene expression and serum TAG between diets and consistent with the findings of others that β-oxidation is decreased in response to dietary ID, the mRNA expression of *Cpt1* was found to be significantly reduced in both the 76- and 93-ID groups. The expression of the iron-containing enzyme *Scd1* was also decreased in the 76-ID group, with the same trend being observed in the 93-ID group. Importantly, expression of the mRNA encoding for the iron uptake protein *Tfr1* was significantly increased while expression of the iron sensor *Hamp1* was significantly decreased in both ID groups. Furthermore, the relative abundance of both *Tfr1* and *Hamp1* mRNA was not different between diets for either the PF or ID animals indicating a similar response to dietary ID, regardless of basal diet. No differences in serum iron or non-heme liver iron between the ID groups on either diet also show that a similar degree of iron deficiency was induced in both ID groups. The moderate level of iron deficiency achieved in this study may serve as an explanation for the differences noted in lipogenic gene expression and serum TAG. Previous work investigating the metabolic lipid response to varying degrees of iron deficiency on an AIN-93 based diet only observed a significant increase in serum TAG at hemoglobin ≤ 66 g/L, a level which is consistent with data suggesting that the severity of metabolic responses to iron deficiency is most highly associated with reductions in hemoglobin [2,4,8,12,35]. Interestingly, despite a more modest induction of iron deficiency for the current study (hemoglobin = 80 g/L), relative elevations in serum glucose and insulin levels were still observed in both the 76-ID and 93-ID groups.

Our findings support the work of others that have clearly demonstrated that even a moderate induction of iron deficiency is sufficient to disrupt normal glucose homeostasis in rodents [2,3,8-10,12,26]. To date, relatively little evidence is available for humans, but an association between ID and elevated hemoglobin A1C (HbA1c) levels has been observed [36]. Indeed, as iron status is improved, levels of HbA1c return to more normal levels [36]. Interestingly, despite what some attribute to so-called over-nutrition, ID is also commonly observed in overweight and obese individuals, a population in which metabolic homeostasis is often disrupted [37-39]. The potential for ID to contribute to or exacerbate conditions wherein normal glucose and fat metabolism are already impaired will be the focus of future investigations.

Currently, the specific mechanisms contributing to iron deficient-induced hyperglycemia remain elusive, but the findings presented herein support the hypothesis that a depletion of iron status, regardless of dietary carbohydrate source, is sufficient to disrupt systemic glucose homeostasis in a weanling rat model of iron deficiency. Intriguingly, enhanced expression of the lipogenic genes *Srebp1c*, *Acly*, and *Fas* was only observed in the ID-76 group. The extent to which these results are suggestive of an iron-level by carbohydrate (i.e., sucrose) interaction remains unknown and is the focus of future studies. To this end, it will also be important to closely examine the changes in nutrient sensing and insulin signaling that occur in response to dietary iron deficiency across different tissues such as the liver and skeletal muscle. The significant increase in *Gck* expression in both ID groups provides some of the first insight into the means by which glucose uptake and utilization is altered in response to iron deficiency [2,9,26]. Future work should focus on the biological significance of this and the other somewhat modest transcriptional changes that occurred with particular attention to the allocation of glucose to various pathways.

## Conclusions

Alterations in serum glucose and insulin observed in ID animals are likely to result from an impaired iron status, rather than the altered utilization of dietary carbohydrate. In contrast, the alterations observed in lipid metabolism (e.g., lipogenic gene expression and serum TAG) in response to ID may be explained by metabolic partitioning of the excess sucrose (relative to AIN-93) present in the AIN-76 diet.

## Abbreviations

AIN: American Institute of Nutrition; C: Control; ID: Iron deficient; PF: Pair fed; TAG: Triacylglycerol; Gck: Glucokinase; Acly: ATP-citrate lyase; Aco1: Aconitase 1; Phdb: Pyruvate dehydrogenase (lipoamide) beta; Pgm3: Phosphoglucomutase 3; Rpia: Ribose 5-phosphate isomerase A; Gys1: Glycogen synthase 1; Gys2: Glycogen synthase 2; Bpgm: 2,3-bisphosphoglycerate mutase; Pdk4: Pyruvate dehydrogenase kinase-4; Cpt1: Carnitine palmitoyltransferase; Fas: Fatty acid synthase; Scd1: Stearoyl-CoA desaturase; Srebp1c: Sterol regulatory element binding protein-1c; Tfr1: Transferrin receptor 1; Hamp1: Hepcidin.

## Competing interest

McKale R. Davis, Kristen K. Hester, Krista M. Shawron, Edralin A. Lucas, Brenda J. Smith, and Stephen L. Clarke have no competing interests.

## Authors’ contributions

MRD and SLC designed the research. MRD, KKH, KMS and SLC conducted the research. MRD, KKH, EAL, BJS, and SLC collected and analyzed data. MRD, BJS, EAL, and SLC wrote the paper. All authors read and approved the final manuscript.

## Supplementary Material

Additional file 1 **Table S1.** Gene Symbols, NCBI Accession Numbers, and Primer Sequences for qPCR. Click here for file
