# Supplementary material for: Comparisons of the iron deficient metabolic response in rats fed either an AIN-76 or AIN-93 based diet
Source: Nutr Metab (Lond). 2012 Oct 30;9:95. doi: 10.1186/1743-7075-9-95 (PMC3538620; doi:10.1186/1743-7075-9-95)
Supplement: Additional file 1 — Table S1. Gene Symbols, NCBI Accession Numbers, and Primer Sequences for qPCR. [file 1743-7075-9-95-S1.docx]

**SUPPLEMENTAL TABLE S1: Gene Symbols, NCBI Accession Numbers, and Primer Sequences for qPCR**

| **Gene Symbol** | **Accession Number** | **Forward Primer** | **Reverse Primer** |
| --- | --- | --- | --- |
| *Gck* | NM_012565 | 5'cggttcaagaatggcatcatc | 5'tcacacccaccaccacgat |
| *Pdk4* | NM_053551 | 5'ggattactgaccgcctctttagtt | 5'gcattccgtgaattgtccatc |
| *Acly* | NM_016987 | 5'tggaggcagcattgcaaa | 5'tctcacaatgcccttgaaggt |
| *Srebp1c* | XM_213329 | 5'gcaacactggcagagatctacgt | 5'tggcgggcactacttaggaa |
| *Fas* | NM_017332 | 5'ggaggaggcggcttctgt | 5'gctgaatacgaccacgcactac |
| *Scd1* | NM_139192 | 5'gctgcccctgaggatcttc | 5'ccgggcccattcatatacat |
| *Cpt1* | NM_013200 | 5'cggttcaagaatggcatcatc | 5'tcacacccaccaccacgat |
| *Tfr1* | NM_022712.1 | 5'tcggctacctgggctattgt | 5'ccgcctcttccgcttca |
| *Hamp1* | NM_053469 | 5'tgacagtgcgctgctgatg | 5'ggaattcttacagcatttacagcaga |
